# Cognitive impairment in patients with very late onset schizophrenia-like psychosis

**DOI:** 10.1192/j.eurpsy.2025.1747

**Published:** 2025-08-26

**Authors:** V. Pochueva, I. Kolykhalov

**Affiliations:** 1 FSBSI Mental Health Research Center, Moscow, Russian Federation

## Abstract

**Introduction:**

Very late-onset schizophrenia-like psychosis (VLOSLP) is one of the largest group of mental illnesses in late life after dementia and depression. The question of role of cognitive impairment and the risk of dementia development in this group is still open.

**Objectives:**

To determine the relationship between cognitive impairment and clinical features.

**Methods:**

65 patients (62 woman, 3 man), medium age 72,5 [63,5; 78,5], medium age of onset 69 [62; 76] with schizophrenia (F20), n=23; chronic delusion disorder (F22.81), n=7, organic schizophrenia-like disorder (F06.2), n=7, schizoaffective disorder (F25), n=11, manifesting after 60 years, underwent clinical examination. The assessment was carried out using clinical-psychopathological, psychometric and statistical methods.

**Results:**

Based on clinical and psychopathological features, 3 clinical groups were formed. The group of patients with acute polymorphic symptoms with mental disorganization included 25 patients. The cognitive impairment was the most acute in this group and correlated with psychotic and affective symptoms. It reduced by the 28^th^ day of investigation, but didn’t reach the normative ones, which may indicate the presence of persistent cognitive dysfunction associated with the present neurobiological changes and creates particular concern regarding the development of a neurodegenerative process in this group of patients.

The 2^nd^ group included 30 patients with a predominance of paranoid symptoms with an “age” coloring, which, however, wasn’t the leading plot. Cognitive impairment was less pronounced compared to the first group and was stable in nature, was not associated with the degree of reduction of psychotic and affective symptoms. This allows us to consider it as a predictor of the development of psychosis.

The 3^rd^ group included 10 patients and was characterized by the prevalence of affective and delusional symptoms with acute sensory delirium, pseudodementia at the height of the condition. The degree of cognitive decline at the beginning of the study was comparable with patients in the 2^nd^ group. High correlations were established between the degree of reduction of productive and affective symptoms and the improvement of cognitive functions. A complete reduction of existing disorders was noted by the end of treatment, which may indicate a more congenial prognosis.

**Conclusions:**

This study confirms the results of previous studies on presence cognitive impairment in patients with VLOSLP, and in addition demonstrates specific differences in cognitive profiles depending on the clinical variant of VLOSLP.

**Disclosure of Interest:**

None Declared

